# Women in STEM: opportunity to improve the health of women and their community

**DOI:** 10.1016/j.lanwpc.2024.101039

**Published:** 2024-02-29

**Authors:** The Lancet Regional Health – Western Pacific

The International Day of Women and Girls in Science is observed on February 11 every year to advance equal access and involvement of women in the fields of science, technology, engineering, and maths (STEM). Globally, only one-third of scientific researchers are women. The UN Secretary-General António Guterres' message on this event emphasised gender equality in science as imperative for a healthy future for all. Equal participation of women and girls in science is vital “to ensure that science works for everyone”. This is particularly true for issues affecting women's health, which affect society as a whole.

The existing biases separating women's health from that of society as a whole, including under-representation in leadership, are major barriers to improving women's health. According to UNESCO's Director-General, Audrey Azoulay, “while women are excluded from leading the global climate agenda, they are the ones who suffer the most from the climate crisis.” In COP28, 15 out of 133 world leaders were women. It has previously been reported that a mere 1.5% of climate-related development aid recognised gender equality as a primary aim, and 0.2% of this aid is received by organisations for women or led by women. The UN Women delegation to COP28 released the Feminist Climate Justice paper which calls for gender-responsive climate policies. An estimated 80% of people displaced due to climate change are women and girls. This may compound pre-existing structure and power inequalities that promote traditional gender-based norms and stereotypes, and place women and girls at greater risk of gender-based violence.

Intimate partner violence (IPV) is the most common type of gender-based violence and contributes significantly to the burden of mental disorders worldwide. Across Asia and the Pacific, over 33% of women report having experienced physical or sexual violence by an intimate partner in their lifetime, 6% higher than the average reported worldwide. Lowe and colleagues found that several individual, relationship, household, and community factors contributed to women's experience of IPV in Samoa, particularly women's employment. Being in paid and unpaid work was protective against IPV and may be related to having more negotiating power and higher status as well as greater means to leave a violent relationship. As such, issues such as IPV, along with poverty, negatively affects educational access and attainment among women and girls, and further exacerbate the shortage of women in health care as well as labour market outcomes and earnings.

An estimated 29% of people working in STEM fields are women, significantly lower than in non-STEM professions (49%). The under-representation of women in STEM careers is especially worrying given that STEM is considered to be the “jobs of the future”, leading innovation, inclusivity, and sustainable development. A staggering 94% of maternal deaths occur in low-resource settings. The majority of these deaths can be attributed to lack of access to skilled health-care providers and services. According to Vallely and colleagues, available data from the Pacific Islands countries and territories (PICTs) suggest that Papua New Guinea has one of the highest maternal mortality ratios, 145–434 per 100,000 live births, far from the SDG 3.1 target of less than 70 per 100,000 live births by 2030. Many of the PICTs continue to experience a workforce shortage in the fields of reproductive, maternal, newborn, child, and adolescent health, especially Papua New Guinea, where the issue has only been made worse by the COVID-19 pandemic and an ageing workforce of nurses and midwives. In Laos, where midwives provide most of the primary maternal and child services, only 21% of these services could be lawfully performed by midwives in primary care settings without a physician.

Greater focus on initiatives and research investigating the impact of sex-specific psychosocial and socioeconomic factors contributing to the physical and mental health of women and girls is fundamental. The *Lancet* women and cardiovascular disease Commission advocated for culturally sensitive and population-based and public policy-based strategies to empower women to further draw upon their agency to make decisions about their own health. Through exploration of the gendered experience of Pacific women health-care workers, Phillips and colleagues found that women health-care workers faced challenges with negotiating professional and caregiving responsibilities and that those in leadership positions positively influenced their colleagues' self-efficacy to handle stresses related to the pandemic. Women's career progression could be facilitated by workplace initiatives that support women with maintaining work and family balance as discussed by Sabarwal and colleagues in a study published in *The Lancet Regional Health-Southeast Asia*. Initiatives supporting women in the region include the Gender-Based Violence Counsellor Training Package for the Pacific, rolled out by the Pacific Women's Network Against Violence Against Women, and the Elimination Partnership in the Indo-Pacific for Cervical Cancer, one of the largest Australian and international collaborations aimed at eliminating cervical cancer in the Pacific.

The International Day of Women and Girls in Science not only celebrates the invaluable contribution women have made and can make in science, but also the importance of women's health and its inextricable link to the health of society as a whole. The health needs of women and girls are interconnected with their unique culture and social setting. We need to break systemic silos that impinge successful solutions and create a more enabling environment for women and girls to equally be at the forefront of research and innovations that meet their health needs and those of their community.

